# Targeting Mutated p53 Dependency in Triple-Negative Breast Cancer Cells Through CDK7 Inhibition

**DOI:** 10.3389/fonc.2021.664848

**Published:** 2021-05-24

**Authors:** Jingyu Peng, Ming Yang, Ran Bi, Yueyuan Wang, Chunxi Wang, Xue Wei, Zhihao Zhang, Xiao Xie, Wei Wei

**Affiliations:** ^1^ Department of Breast Surgery, The First Hospital of Jilin University, Changchun, China; ^2^ Key Laboratory of Organ Regeneration & Transplantation of the Ministry of Education, Institute of Translational Medicine, The First Hospital of Jilin University, Changchun, China; ^3^ Department of Urology, The First Hospital of Jilin University, Changchun, China

**Keywords:** CDK7 inhibition, THZ1, triple-negative breast cancer, mutated p53, targeted therapy, cancer treatment

## Abstract

**Background:**

Cyclin-dependent kinase 7 (CDK7) is crucial for cell cycle progression and gene expression transcriptional regulation, which are often not assessed in cancer developing process. CDK7 inhibitors have emerged as promising drugs for treating diverse cancers, including breast cancer. However, the mechanism behind its anticancer effect has not been well investigated. Here, the possible mechanism of CDK7 inhibitors for treating human triple-negative breast cancer (TNBC) has been studied.

**Methods:**

The effects of CDK7 inhibitors on breast cancer cells have been identified by measuring cell viability (Cell Counting Kit-8) and cell proliferation and calculating colony formation. The short hairpin RNA and short interfering RNA were used for the construction of knockdown cells. To assess the expression of associated proteins, western blot was used.

**Results:**

This study confirmed that, compared to hormone receptor-positive breast cancer cells, TNBC cells were more sensitive to THZ1, a novel CDK7 inhibitor. THZ1 treatment specifically downregulated mutated p53 in a dose- and time-dependent manner in TNBC cells with p53 mutation. Another CDK7 inhibitor, LDC4297, also potently interfered with the expression of mutated p53. Furthermore, endogenous CDK7 expression was positively correlated with the levels of mutated p53 in TNBC cells with p53 mutation. Downregulating mutated p53 expression significantly suppressed the proliferation of TNBC cells with p53 mutation.

**Conclusion:**

Our findings demonstrated that targeting CDK7 was an effective approach for the treatment of TNBC with p53 mutation.

## Introduction

Triple-negative breast cancer (TNBC), a heterogeneous breast cancer subtype, lacks endocrine estrogen receptor (ER) and progesterone receptor (PR) and human epidermal growth factor receptor 2 (HER2, encoded by ERBB2) (1, 2). When comparing to other breast cancer subtypes, TNBC is more likely to recur and develop resistance to endocrine or anti-HER2 therapy (1, 3). Unfortunately, although targeted drugs such as poly (ADP)-ribose polymerase inhibitors have been applied for TNBC therapy, the scope and efficacy of its application are limited. Currently, chemotherapy is still the main systemic treatment for TNBC patients. Although it initially responds well to chemotherapy, TNBC remains to have a poorer prognosis than non-TNBC. For stage I triple-negative tumors, the 5-year breast cancer-specific survival rate is 85%, while that of ERBB2 and hormone receptor-positive cancer ranges from 94% to 99% (4). Hence, there is an urgent need for therapeutic approaches against TNBC.

Pharmacological blockade of transcription is a potential anticancer strategy. Some members of the cyclin-dependent kinases (CDKs), such as CDK7, CDK9, CDK12, and CDK13, which regulate transcription by phosphorylating RNA polymerase (RNAP) II, are possible drug targets (5). CDK7, an essential component of CDK-activating kinase (CAK) and the general transcription factor IIH (TFIIH), modulates cell division cycle and transcription (6). CDK7 binds with two other subunits (cyclin-H and MAT1) to form the complex CAK mediating the activation of other CDKs, including CDK1, CDK2, CDK4, and CDK6 (7). Besides, CDK7 is also one component of TFIIH that phosphorylates RNAP II or other transcription factors, such as androgen receptor and ER (8). Thus far, accumulating studies have demonstrated that CDK7 is frequently overexpressed in diverse tumor tissues including gastric cancer, oral squamous cell carcinoma, and breast cancer (9–12). Preclinical studies have confirmed that covalent CDK7 inhibitors selectively induce apoptosis in certain cancers, but not in normal human cells (13–18).

THZ1 is a phenylamino-pyrimidine that covalently targets CDK7 by covalently binding to its adenosine triphosphate (ATP) site allosterically with strong antitumor activity against TNBC cells (19). However, its effects on ER/PR+ breast cancer cells still remain controversial (19, 20). Moreover, the mechanism behind its anticancer activity has not yet been described. In this study, we investigated whether TNBC cells were more susceptible than ER/PR+ breast cancer cells to THZ1 therapy. Our results indicated that THZ1 selectively downregulated endogenous mutant p53 levels in TNBC cells with p53 mutation, but not in other breast cancer cells. Another CDK7 inhibitor treatment (LDC4297, a competitive inhibitor of ATPase) or silencing of CDK7 expression also decreased mutant p53 in TNBC cells with p53 mutation. Direct downregulation of mutant p53 expression levels effectively suppressed the proliferation of TNBC cells with p53 mutation. These results indicate the potential usage of CDK7 inhibitors in the treatment of TNBCs with p53 mutant by downregulating the content of endogenous mutant p53.

## Materials and Methods

### Cell Culture and Compound Preparation

Hs-578T (TNBC cells with mutant-type p53) and MCF-7 (non-TNBC cells with wild-type p53) were 10% fetal bovine serum cultivated in Dulbecco’s Modified Eagle’s medium (Biological Industries, USA) from the Type Culture Set of the Chinese Academy of Sciences (Shanghai, China). DU4475 (TNBC cells with wild-type p53) were 10% fetal bovine serum cultivated in 1640 medium (Biological Industries, USA) from the FuHeng BioLogy (Shanghai, China). Under normal incubator conditions (37°C and 5% carbon dioxide), all cells were developed. THZ1 (HY-80013) and LDC4297 (HY-126p53) were obtained from MedChemExpress (Monmouth Junction, NJ, USA). All drugs we used in this article were dissolved in a stock solution of 10 mM dimethyl sulfoxide (DMSO).

### Assay of Cell Viability

To detect cell viability, we chose the Cell Counting Kit-8 (CCK-8, TransGen Biotech) and performed the assay following the manufacturer’s instructions. Nearly 8,000 cells were seeded onto 96-well plates per well and incubated until detection for 24 h. We established three parallel groups for each test group. Cells were subsequently treated with different THZ1 concentrations for 48 h. The cells were then incubated in 100 μL of cell culture medium containing 10 μL CCK-8 for 2 h at 37°C. The absorbance was observed using the BioTek ELISA reader (Winooski, VT, USA) at a wavelength of 450 nm. Cell inhibitory ratio was calculated using the following formula:

100×cell growth inhibitory ratio(%)=100×([A×450control−A450sample][A×450control−A450blank])

### Microscopy Images

Cells (approximately 1 × 10^5^) were planted in 12-well plates and treated for 48 h at different concentrations of THZ1 or vehicle control (DMSO) or treated with short interfering RNAs (siRNAs) (si-889) that target the human p53 gene and negative control siRNAs (si-con) for 48 h. Static bright-field images were photographed using OLYMPUS cellSens Standard software (Olympus).

### Cell Proliferation Assay

For growth assays, Hs-578T (4 × 10^4^), DU4475(8 × 10^4^), or MCF-7(5 × 10^4^) cells were seeded into 12-well plates and were then treated for 24 h and 48 h with THZ1 or DMSO (as control) or treated with siRNAs (si-889) that target the human p53 gene and negative control siRNAs (si-con) for 24 h and 48 h. We established three parallel groups for each test group. Cells were counted using the cell counting chamber (Shanghai Precision Instruments Co., Ltd., Shanghai, China) at specified time points and staining with trypan blue. We repeated all of the tests three times.

### Cell Colony Formation Assays

In colony formation assays, approximately 5000 cells/well were plated with 2-mL medium in 6-well plates, treated with THZ1 or DMSO (as control) in different concentrations or treated with siRNAs (si-889) that target the human p53 gene and negative control siRNAs (si-con), and incubated for 7 days. Cells were fixed with 100% methanol for 30 min and stained with 20% ethanol with 0.2 g/mL crystal violet solution after washing with cold phosphate-buffered solution (PBS) twice. Phase-contrast microscopy (Olympus) was used to detect colonies, and colonies not less than 50 cells were counted. We repeated all of the tests three times. Because DU4475 cells are cells that grow in suspension, it is inconvenient to perform colony formation assays; thus, this experiment did not involve corresponding content.

### Western Blot

Until lysing cells in the RIPA buffer (Beyotime, China), cells were washed twice with cold PBS, followed by incubation for 10 min at 4°C. At 12,000 g at 4°C, the whole-cell lysate was centrifuged and the supernatant was collected, mixing with the loading buffer. Then, it was denatured for 10 min at 100°C, followed by electrophoresis of 12% sodium dodecyl sulfate polyacrylamide gel. The isolated proteins were transferred to membranes of nitrocellulose (Bio-Rad, USA). The membranes were probed overnight with sufficiently diluted primary antibodies after being blocked with 5% nonfat milk. After washing the membranes, they were incubated for 1 h at room temperature with alkaline phosphatase-conjugated secondary antibodies. The protein bands were visualized by 5-bromo-4-chloro-3-indolyl phosphate and nitro blue tetrazolium (Millipore). Western blotting images were acquired on a scanner (Epson Perfection V330 Photo) using Scan-n-Stitch Deluxe software (version 1.1.9, ArcSoft). Quantification analysis of protein expression was analyzed using ImageJ software (version 1.8.0, National Institutes of Health, Bethesda, MD, USA).

The primary antibodies included CDK7 (cat# 2916T, Cell Signaling Technology), p53 (cat# 10442-1-AP, Proteintech), GAPDH (cat# 10494-1-AP, Proteintech), and tubulin (cat# 11224-1-AP, Proteintech). Secondary antibodies included anti-rabbit IgG (cat# 7054) and anti-mouse IgG (cat# 7056) (Cell Signaling Technology, USA). All experiments were repeated three times.

### Preparation of Lentiviruses

pRSV-Rev (122p53), pMDLg/pRRE (12251), and pCMV-VSV-G (8454) were bought from Addgene (Cambridge, MA, USA). The short hairpin RNAs (shRNAs) (5′-CCGGGCTGTAGAAGTGAGTTTGTAACTCGAGTTACAAACTCACTTCTACAGCTTTTT-3′) that target human CDK7 (sh-CDK7) were purchased from Sigma-Aldrich (USA). Viruses were produced by 293T cells. After transfection, the supernatant of 293T cells was collected for 48 h and then purified through 0.45-μm membranes. Puromycin (1.5 μg/mL) was used for 2 days to pick cells for selection.

### Short Interfering RNAs for Knockdown Cell Building

The siRNAs (si-889) (5′-CCACCAUCCACUACAACUATT-3′), which target the human p53 gene, and negative control siRNAs (si-con) were obtained from GenePharma (Shanghai, China). Lipofectamine 3000 (Thermo Fisher Scientific, USA) was used to conduct the cellular delivery of siRNA into Hs-578T and MCF-7 cells. jetPRIME (Polyplus-transfection, France) was used to conduct the cellular delivery of siRNA into DU4475 cells. Approximately 6000 Hs-578T and MCF-7 cells/well and 15000 DU4475 cells/well, as instructed by the manufacturer, were seeded into 12 wells after transfection with siRNAs or negative control. After 48 h, a part of the cells was collected for testing the gene interference ability of siRNA, and the other part of the cells was used for subsequent proliferation assay and clone formation assay.

### Statistical Analyses

Using GraphPad Prism 6 (GraphPad Software, CA, USA), all data were analyzed and presented as the mean ± standard deviation. The differences between the two groups were compared by t-test, and the difference between multiple groups was compared using analysis of variance. P < 0.05 was considered statistically significant.

## Results

### Distinct Efficiency of THZ1 on the Growth Inhibition of Triple-Negative Breast Cancer (TNBC) and Estrogen Receptor+ Breast Cancer Cells

**Figure 1:**
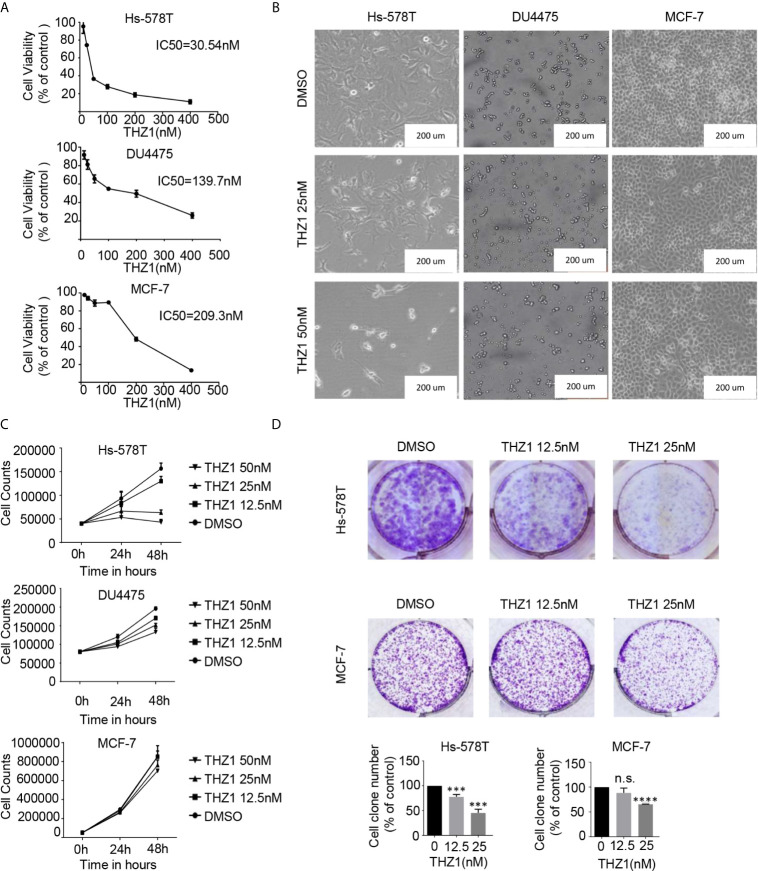
THZ1 inhibits cell proliferation in breast cancer cells. **(A)** Cell Counting Kit-8 assays have determined the proliferation of breast cancer cells after treatment with dimethyl sulfoxide (DMSO) (control) or indicated concentrations of THZ1 (12.5, 25, 50, 100, 200, and 400 nM) for 48 h. **(B)** Images (200×) show Hs-578T, DU4475, and MCF-7 cells following THZ1 treatment for 48 h. **(C)** Effects of THZ1 treatment on breast cancer cell proliferation curve. Verification was performed by cell counting. **(D)** Impact of treatment with THZ1 on the development of breast cancer cells in the colony. Cells have been treated with either DMSO (control) or indicated THZ1 concentrations (12.5, 25, and 50 nM) for 7 days. With an inverted microscope, cell colonies (>50 cells) were counted. The mean ± standard deviation reflects the data. (n.s., non significant, P > 0.05; ^***^P < 0.001, ^****^P < 0.0001, n = 3).

Recent mRNA expression profiling and immunohistochemistry studies have documented elevated CDK7 expression in breast cancer tumors (10). Previous studies have investigated the correlations between CDK7 RNA expression and relapse-free survival (RFS) in breast cancer using a microarray database of 3,951 breast cancer patients and found that high expression levels of CDK7 are closely associated with worse RFS in all breast cancer subtypes (20). To illustrate the effect of THZ1 on breast cancer, we profiled several groups of cell lines representing various subtypes of breast cancer. The cell viability assay indicated that the 50% inhibitory concentration values of THZ1 in Hs-578T (TNBCs with mutant-type p53), DU4475 (TNBCs with wild-type p53), and MCF-7 (ER+) cells were 30.54 nM, 139.7 nM, and 209.3 nM, respectively (**Figure 1A**). This indicated that different breast cancer cells had variable sensitivity to THZ1. This was further supported by the results showing that 50 nM of THZ1 did not cause significant suppression of DU4475 cell and MCF7 cell proliferation (**Figures 1B, C**). Next, we performed colony formation assays, and the results showed that THZ1 inhibited breast cancer proliferation chronically and effectively. ER+ MCF-7 cells were significantly less sensitive to THZ1 compared to TNBC cells (**Figure 1D**). These findings demonstrated that THZ1 was effective in multiple subtypes of breast cancer, but its efficiency varies depending on the subtype.

### THZ1 Selectively Suppressed The Expression of Mutated p53 in TNBC Cells With p53 Mutation

**Figure 2:**
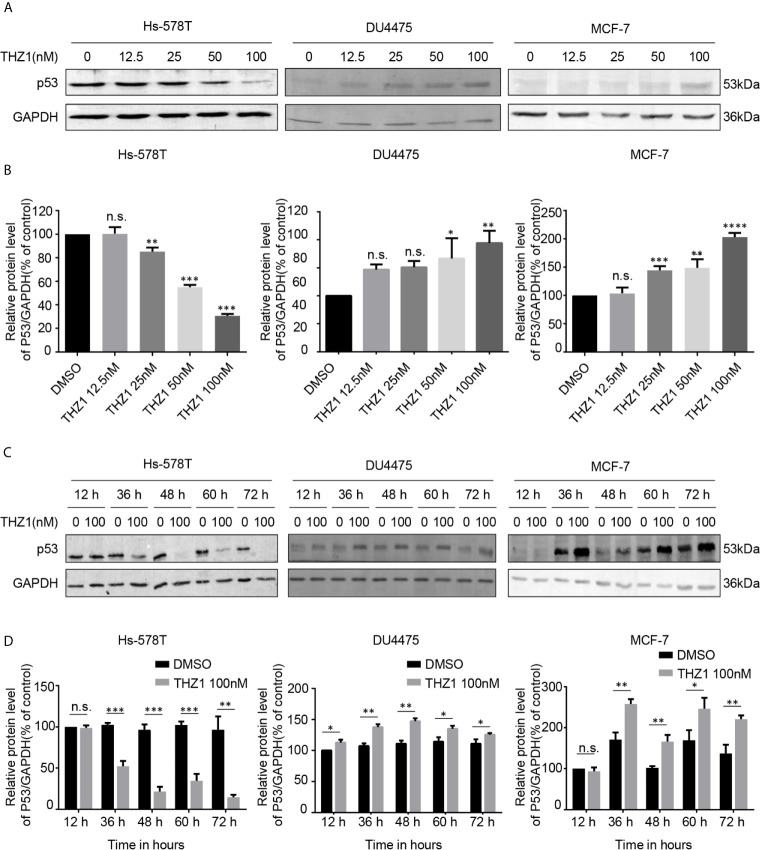
THZ1 downregulates the expression of mutant-type p53 rather than wild-type p53 in breast cancer cells. **(A)** Western blot was used to detect relative p53 protein expression in breast cancer cells after treatment with dimethyl sulfoxide (DMSO) (control) or specified concentrations of THZ1 (12.5, 25, 50, and 100 nM) for 48 h. **(B)** Quantification analysis of p53 protein expression after treatment with DMSO (control) or indicated concentrations of THZ1 (12.5, 25, 50, and 100 nM) for 48 h. **(C)** Western blot analysis of relative p53 expression levels in breast cancer cells was performed at different times(12 h, 36 h, 48 h, 60 h, and 72 h) following treatment with DMSO (control) or the same THZ1 concentration. **(D)** Quantification analysis of p53 protein expression after treatment with DMSO (control) or the same concentration of THZ1(100 nM) for different durations (12 h, 36 h, 48 h, 60 h, and 72 h). The mean ± standard deviation reflects the data. (n.s., non significant, P > 0.05; ^*^P < 0.05, ^**^P < 0.01, ^***^P < 0.001, ^****^P < 0.0001, n = 3).

**Figure 3:**
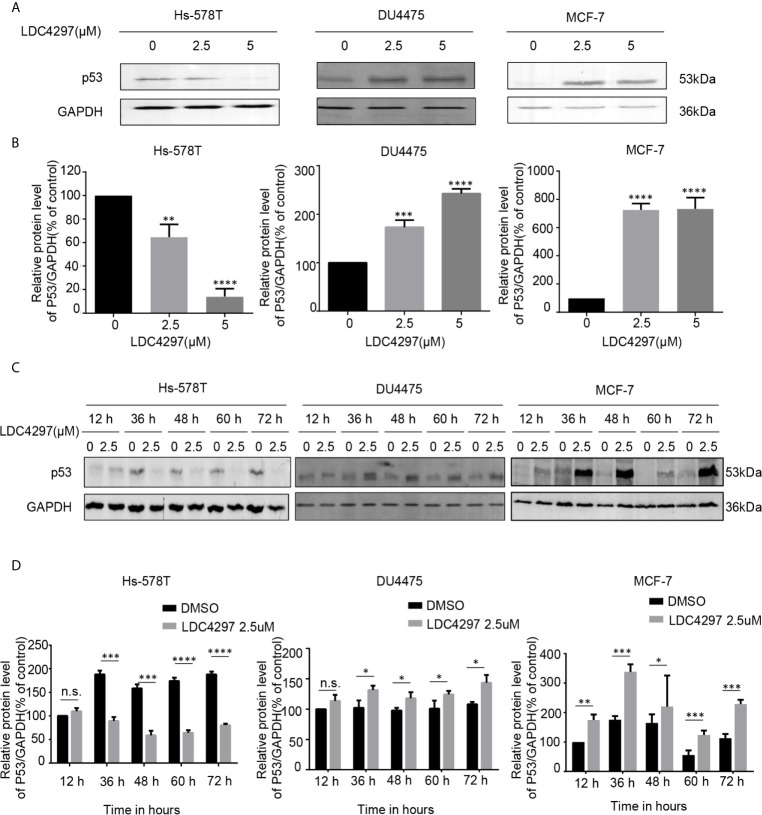
The expression of mutant-type p53 instead of wild-type p53 is downregulated after the expression of cyclin-dependent kinase 7 is blocked by LDC4297. **(A)** Western blot was used to detect relative p53 protein expression in breast cancer cells after treatment with dimethyl sulfoxide (DMSO) (control) or specified concentrations of LDC4297 (2.5 and 5 uM) for 48 h. **(B)** Quantification analysis of p53 protein expression after treatment with DMSO (control) or indicated concentrations of LDC4297 (2.5 and 5 uM) for 48 h. **(C)** The relative expression levels of p53 in breast cancer cells were analyzed by Western blot after treatment with DMSO (control) or the same concentration of LDC4297 (2.5 uM) for different times(12–72 h). **(D)** Quantification analysis of p53 protein expression after treatment with DMSO (control) or the same concentration of LDC4297 (2.5 uM) for different times (12–72 h). The mean ± standard deviation reflects the data. (n.s., non significant, P > 0.05; ^*^P < 0.05, ^**^P < 0.01, ^***^P < 0.001, ^****^P < 0.0001, n = 3).

The exact mechanism of the determinants of the sensitivity of cancer cells to THZ1 has not yet been characterized. We observed that TNBC cells bearing genetic p53 mutations were more sensitive to THZ1. Interestingly, we found that THZ1 potently decreased p53 protein expression in TNBC cells with p53 mutations (Hs-578T cells) in a dose-dependent manner. However, this effect was not observed in TNBC cell lines with wild-type p53 (DU4475 cells) or ER-positive MCF-7 cells with wild-type p53 (**Figures 2A, B**). We also confirmed that the suppression of mutated p53 expression in TNBC cells by THZ1 was time-dependent (**Figures 2C, D**). Next, we used another CDK7 inhibitor, LDC4297, to further verify the effect of CDK7 inhibitors on p53 protein expression. Similarly, LDC4297 treatment effectively downregulated p53 expression in TNBC cells with mutant-type p53 (Hs-578T), but not in TNBC cell lines with wild-type p53 (DU4475 cells) or ER-positive MCF-7 cells with wild-type p53. Moreover, the manner of its suppressive effect was dependent on dose and time (**Figure 3**). Hence, we found that CDK7 inhibitors selectively interfered with mutated p53 expression in TNBC cells with p53 mutation, but they did not influence the expression of wild-type p53 in other breast cancer cells.

### Cyclin-Dependent Kinase 7 Is Critical for the Expression of Mutated p53 in TNBC Cells With p53 Mutation

**Figure 4:**
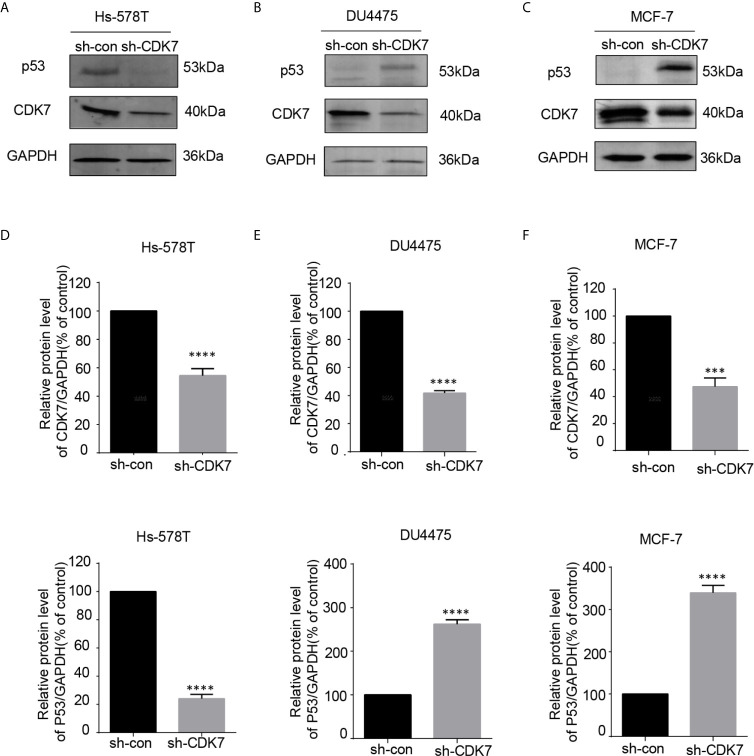
The expression of mutant-type p53 instead of wild-type p53 is downregulated after the expression of cyclin-dependent kinase 7 (CDK7) is blocked by sh-CDK7. **(A–C)** The relative expression of CDK7 and p53 protein detected by western blot in Hs-578T, DU4475, and MCF-7 cells, with CDK7 being knocked down by short hairpin RNA. **(D–F)** Quantification analysis of CDK7 and p53 protein expression. The mean ± standard deviation reflects the data. (^***^P < 0.001, ^****^P < 0.0001, n = 3).

Since CDK7 inhibitors interfered with mutated p53 expression, we proceeded to investigate the effects of CDK7 proteins on the regulation of mutated p53 in breast cancer cells. We generated CDK7 stable knockdown Hs-578T, DU4475, and MCF-7 cells using shRNA targeting CDK7 (**Figures 4A, C**). The immunoblotting data indicated that p53 expression levels were dramatically downregulated in CDK7 knockdown Hs-578T cells, but not in sh-CDK7 DU4475 and MCF7 cells (**Figure 4**). These results confirmed that CDK7 supported mutated p53 expression in TNBC cells with p53 mutation.

### Mutated p53 Proteins Support the Proliferation of TNBC Cells With p53 Mutation

**Figure 5:**
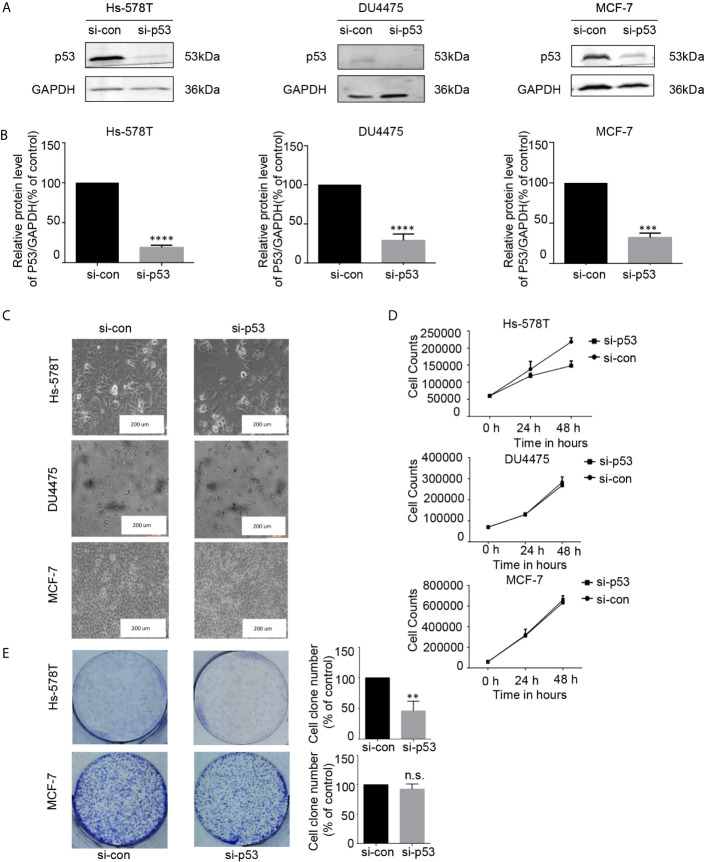
The proliferation of breast cancer cell lines was inhibited after the expression of p53 protein was downregulated. **(A, B)** Effects of p53 knockdown using short interfering RNA (siRNA) in breast cancer cell lines. **(C)** Images (200×) show breast cancer cells after 48 h of siRNA treatment **(D)** Effects of siRNA treatment on breast cancer cell line proliferation curve. Verification by cell counting. **(E)** Impact of treatment with p53 knockdown on the colony formation of breast cancer cells. Cell colonies (>50 cells) were counted. The mean ± standard deviation reflects the data. (n.s., non significant, P > 0.05; ^**^P < 0.01, ^***^P < 0.001, ^****^P < 0.0001, n = 3).

THZ1 specifically decreased mutated p53 expression in TNBC cells with p53 mutation. This suggested that the growth inhibitory activity of THZ1 in TNBC cells with p53 mutation relied on mutated p53 downregulation. We used RNA interference to downregulate endogenous p53 expression in TNBC or ER+ breast cancer cells (**Figures 5A, B**). Morphological changes in si-p53 and control cells suggested that mutated p53 proteins were essential for the survival of Hs-578T cells, but not DU4475 and MCF-7 cells (**Figure 5C**). Cell proliferation and colony formation assays further supported the fact that knockdown p53 inhibited the proliferation of TNBC cells with mutant p53, but not TNBC or ER+ cells with wild-type p53 (**Figures 5D, E**). Our data clarified the determinant roles of p53 mutant for the maintenance of cell survival of p53-mutated TNBCs.

## Discussion

CDK7 plays a dual role in driving cell cycle progression and modulating transcription (6). Its expression frequently increases in many cancers and is important for tumor development (10, 12). A number of CDK7 inhibitors are considered to be potential drug candidates for cancer therapy because they are highly cytotoxic to tumor cells only (13, 21, 22). One main concern regarding the application of CDK7 inhibitors in cancer therapy is the variable sensitivity of different cancer types to CDK7 inhibition, which has been found in preclinical studies. In this study, we selected drugs affected through different pathways to inhibit CDK7 and established adenovirus containing vectors with CDK7-shRNA to interfere its expression. Fortunately, we identified TNBC cells with p53 mutations as most sensitive to CDK7 inhibitors because mutated p53-dependent cancer cell proliferation was selectively inhibited.

Previous studies have demonstrated that TNBC cells particularly rely on CDK7 due to a group of CDK7 kinase-controlled TNBC-specific genes (19). Other experiments have shown that no apparent selectivity between different subtypes of breast cancer under CDK7 treatment was detected (20). In addition, not only recent mRNA expression profiling but also immunohistochemistry studies have demonstrated that the expression of CDK7 in ER+ breast cancer was elevated (10). By comparing the results after adding varied THZ1 concentrations to different cell lines of breast cancer, we confirmed that THZ1 has a broad antitumor activity with diverse sensitivities. We further explored the mechanisms that contributed to the different sensitivities of different breast cancer cells to CDK7 inhibition. This study also emphasized the importance of p53 status in breast cancer cells for CDK7 inhibitor treatment.

TNBC has a greater level of genetic sophistication compared to the other breast cancer subtypes, as shown by its higher prevalence of point mutation, gene amplification, and deletion (3). Since TNBC is ER/PR- and HER2-negative, common drugs used for breast cancer therapies such as tamoxifen; letrozole, which targets hormone receptors; and trastuzumab, which aims to destroy HER2 overexpression in breast cancer cells, are inapplicable in TNBC treatment. Researchers are consistently identifying several drugs designed to suppress TNBC development. A mutated p53 tumor suppressor gene is found in over 80% of TNBC cells (23–25). Previous studies have focused on reactivating mutant p53 and converting it into a wild-type mutant *via* small molecule inhibitors. Their results showed that this approach efficiently suppressed TNBC cell growth (26, 27). However, there are a variety of mutation sites and multiple mutation forms for mutant p53; thus, reactivating p53 might not lead to a satisfied cost performance. Hence, we suggest that decreased mutant-type p53 protein expression would be a distinct idea for TNBC with mutant-type p53 treatment. This study shows that the use of CDK7 inhibitors to directly downregulate the content of endogenous mutant-type p53 protein can inhibit the proliferation of p53 mutant-type TNBC cells. These findings support the fact that mutated p53 is a determinant factor for the survival of TNBC cells with p53 mutation.

Cell cycle progression by CDKs T-loop phosphorylation is guided by the CAK, comprising CDK7, cyclin H, and MAT1. CDK7 is also a vital member of the general TFIIH (6). In contrast to the specific downregulation of intracellular accumulation of mutated p53 by CDK7 inhibition in TNBC cells, our data also show that CDK7 inhibitor treatment could elevate the expression of p53 in breast cancer cells with wild-type p53 (**Figures 2**, **3**). Recent studies have observed that CDK inhibition elevated the expression of p53 proteins in human colon cancer-derived cells (28). p53 is recognized and phosphorylated by CDK7-cyclinH-p36 trimeric complex (29). Posttranslational phosphorylation of p53 regulates its transcriptional activity and stability. The roles of CDK7, cyclin H, and p36 mediated by p53 phosphorylation on the intracellular accumulation of wild-type and mutant-type p53 in breast carcinoma cells need further investigation in future studies. Besides, all our conclusions are based on the *in vitro* results from the immortalized breast carcinoma-derived cell lines. Relevant experiments that determine the effects of CDK7 inhibitors on the stability and function of mutated p53 *in vivo* are equally important to evaluate the potential usage of CDK7 inhibitors in the therapeutic treatment of TNBC patients with p53 mutant.

In summary, our results suggested that CDK7 facilitates the proliferative activity of TNBC cells with p53 mutation by maintaining high expression of mutated p53. This established a regulatory association between CDK7 kinase activity and mutated p53 expression. Downregulation of p53 by CDK7 inhibition is an optimal drug target for TNBC or even for other carcinomas bearing a p53 mutation.

## Data Availability Statement

The datasets presented in this study can be found in online repositories. The names of the repository/repositories and accession number(s) can be found in the article/**Supplementary Material**.

## Author Contributions

Manuscript writing: JP, WW, MY. Data analysis: YW, ZZ. Study design: JP, RB, CW. Data collection: XW, XX. All authors contributed to the article and approved the submitted version.

## Conflict of Interest

The authors declare that the research was conducted in the absence of any commercial or financial relationships that could be construed as a potential conflict of interest.

## References

[1] Cancer Genome Atlas, Network. Comprehensive Molecular Portraits of Human Breast Tumors. Nature (2012) 490(7418):61–70. 10.1038/nature11412 23000897 PMC3465532

[2] Wang,W Zhang B Mani AM Wu Z Fan Y Li W. Survivin Inhibitors Mitigate Chemotherapeutic Resistance in Breast Cancer Cells by Suppressing Genotoxic Nuclear Factor-κb Activation. J Pharmacol Exp Ther (2018) 366(1):184–93. 10.1124/jpet.118.249151 PMC6038029 29735611

[3] Giampaolo B Balko JM Mayer IA Sanders ME Gianni L. Triple-Negative Breast Cancer: Challenges and Opportunities of a Heterogeneous Disease. Nat Rev Clin Oncol (2016) 13(11):674–90. 10.1038/nrclinonc.2016.66 PMC5461122 27184417

[4] Waks AG Winer EP. Breast Cancer Treatment. Jama (2019) 321(3):288–300. 10.1001/jama.2018.19323 30667505

[5] Chou, J., D. A. Quigley TM Robinson F Feng Y Ashworth A. Transcription-Associated Cyclin-Dependent Kinases as Targets and Biomarkers for Cancer Therapy. Cancer Discovery (2020) 10(3):351–70. 10.1158/2159-8290.CD-19-0528 32071145

[6] Malumbres M. Cyclin-Dependent Kinases. Genome Biol (2014) 15:122. 10.1186/gb4184 25180339 PMC4097832

[7] Fisher RP. The CDK Network: Linking Cycles of Cell Division and Gene Expression. Genes Cancer (2012) 3:731–8. 10.1177/1947601912473308 PMC3636752 23634260

[8] Patel H Abduljabbar R Lai CF Periyasamy M Harrod A Gemma C. Expression of CDK7, Cyclin H, and MAT1 Is Elevated in Breast Cancer and Is Prognostic in Estrogen Receptor-Positive Breast Cancer. Clin Cancer Res (2016) 22:5929–38. 10.1158/1078-0432.CCR-15-1104 PMC5293170 27301701

[9] Li B Ni Chonghaile T Fan Y Madden SF Klinger R O’Connor AE. Therapeutic Rationale to Target Highly Expressed Cdk7 Conferring Poor Outcomes in Triple-Negative Breast Cancer. Cancer Res (2017) 77(14):3834–45. 10.1158/0008-5472.Can-16-2546 28455421

[10] Patel H Abduljabbar R Lai CF Periyasamy M Harrod A Gemma C. Expression of CDK7, Cyclin H, and MAT1 Is Elevated in Breast Cancer and Is Prognostic in Estrogen Receptor-Positive Breast Cancer. Clin Cancer Res (2016) 22(23):5929–38. 10.1158/1078-0432.CCR-15-1104 PMC5293170 27301701

[11] Diab S Yu M Wang S. Cdk7 Inhibitors in Cancer Therapy: The Sweet Smell of Success? J Med Chem (2020) 63(14):7458–74. 10.1021/acs.jmedchem.9b01985 32150405

[12] Kim J Cho YJ Ryu JY Hwang I Han HD Ahn HJ. CDK7 Is a Reliable Prognostic Factor and Novel Therapeutic Target in Epithelial Ovarian Cancer. Gynecol Oncol (2020) 156(1):211–21. 10.1016/j.ygyno.2019.11.004 PMC8861881 31776040

[13] Kwiatkowski N Tinghu Z Peter B Rahl B Abraham J Reddy J. Targeting Transcription Regulation in Cancer With a Covalent CDK7 Inhibitor. Nature (2014) 511(7511):616–+. 10.1038/nature13393 PMC4244910 25043025

[14] Greenall SA Lim YC Mitchell CB Ensbey KS Stringer BW Wilding AL. Cyclin-Dependent Kinase 7 Is a Therapeutic Target in High-Grade Glioma. Oncogenesis (2017) 6(5):e336. 10.1038/oncsis.2017.33 28504693 PMC5523066

[15] Cheng ZJ Miao DL Su QY Tang XL Wang XL Deng LB. THZ1 Suppresses Human non-Small-Cell Lung Cancer Cells In Vitro Through Interference With Cancer Metabolism. Acta Pharmacol Sin (2019) 40(6):814–22. 10.1038/s41401-018-0187-3 PMC6786356 30446732

[16] Lu,P Geng J Zhang L Wang Y Niu N Fang Y. THZ1 Reveals CDK7-dependent Transcriptional Addictions in Pancreatic Cancer. Oncogene (2019) 38(20):3932–45. 10.1038/s41388-019-0701-1 30692639

[17] Lei G Li J Zeng H Guzman AG Li T Lee M. A Combination Strategy Targeting Enhancer Plasticity Exerts Synergistic Lethality Against BETi-resistant Leukemia Cells. Nat Commun (2020) 11(1):740–55. 10.1038/s41467-020-14604-6 PMC7005144 32029739

[18] Huang T Ding X Xu G Chen G Cao Y Peng C. CDK7 Inhibitor THZ1 Inhibits MCL1 Synthesis and Drives Cholangiocarcinoma Apoptosis in Combination With BCL2/BCL-XL Inhibitor ABT-263. Cell Death Dis (2019) 10(8):602. 10.1038/s41419-019-1831-7 31399555 PMC6688996

[19] Wang Y Zhang T Kwiatkowski N Abraham BJ Lee TI Xie S. CDK7-Dependent Transcriptional Addiction in Triple-Negative Breast Cancer. Cell (2015) 163(1):174–86. 10.1016/j.cell.2015.08.063 PMC4583659 26406377

[20] McDermott MSJ Sharko AC Munie J Kassler S Melendez T Lim CU. Cdk7 Inhibition Is Effective in All the Subtypes of Breast Cancer: Determinants of Response and Synergy With EGFR Inhibition. Cells (2020) 9(3):638–55. 10.3390/cells9030638 PMC7140476 32155786

[21] Sava GP Fan H Coombes RC Buluwela L Ali S. CDK7 Inhibitors as Anticancer Drugs. Cancer Metastasis Rev (2020) 39(3):805–23. 10.1007/s10555-020-09885-8 PMC7497306 32385714

[22] Olson CM Liang Y Leggett A Park WD Li L Mills CE. Development of a Selective Cdk7 Covalent Inhibitor Reveals Predominant Cell-Cycle Phenotype. Cell Chem Biol (2019) 26(6):792–803.e10. 10.1016/j.chembiol.2019.02.012 30905681 PMC6588464

[23] Lawrence MS Stojanov P Mermel CH Robinson JT Garraway LA Golub TR. Discovery and Saturation Analysis of Cancer Genes Across 21 Tumour Types. Nature (2014) 505(7484):495–501. 10.1038/nature12912 24390350 PMC4048962

[24] Nik-Zainal S Davies H Staaf J Ramakrishna M Glodzik D Zou X. Landscape of Somatic Mutations in 560 Breast Cancer Whole-Genome Sequences. Nature (2016) 534(7605):47–54. 10.1038/nature17676 27135926 PMC4910866

[25] Silwal-Pandit L Vollan HK Chin SF Rueda OM McKinney S Osako T. TP53 Mutation Spectrum in Breast Cancer Is Subtype Specific and has Distinct Prognostic Relevance. Clin Cancer Res (2014) 20(13):3569–80. 10.1158/1078-0432.CCR-13-2943 24803582

[26] Synnott NC Bauer MR Madden S Murray A Klinger R O’Donovan N. Mutant p53 as a Therapeutic Target for the Treatment of Triple-Negative Breast Cancer: Preclinical Investigation With the Anti-p53 Drug, PK11007. Cancer Lett (2018) 414:99–106. 10.1016/j.canlet.2017.09.053 29069577

[27] Synnott NC Murray A McGowan PM Kiely M Kiely PA O’Donovan N. Mutant p53: A Novel Target for the Treatment of Patients With Triple-Negative Breast Cancer? Int J Cancer (2017) 140(1):234–46. 10.1002/ijc.30425 27615392

[28] Kalan S Amat R Schachter MM Kwiatkowski N Abraham BJ Liang Y. Activation of the P53 Transcriptional Program Sensitizes Cancer Cells to Cdk7 Inhibitors. Cell Rep (2017) 21(2):467–81. 10.1016/j.celrep.2017.09.056 PMC5687273 29020632

[29] Lu H Fisher RP Bailey P Levine AJ. The CDK7-cycH-p36 Complex of Transcription Factor IIH Phosphorylates p53, Enhancing Its Sequence-Specific DNA Binding Activity In Vitro. Mol Cell Biol (1997) 17(10):5923–34. 10.1128/mcb.17.10.5923 PMC232440 9315650

