# Supplementary material for: Targeting Mutated p53 Dependency in Triple-Negative Breast Cancer Cells Through CDK7 Inhibition
Source: Front Oncol. 2021 May 24;11:664848. doi: 10.3389/fonc.2021.664848 (PMC8183379; doi:10.3389/fonc.2021.664848)
Supplement: Supplementary file 1 [file DataSheet_1.pdf]

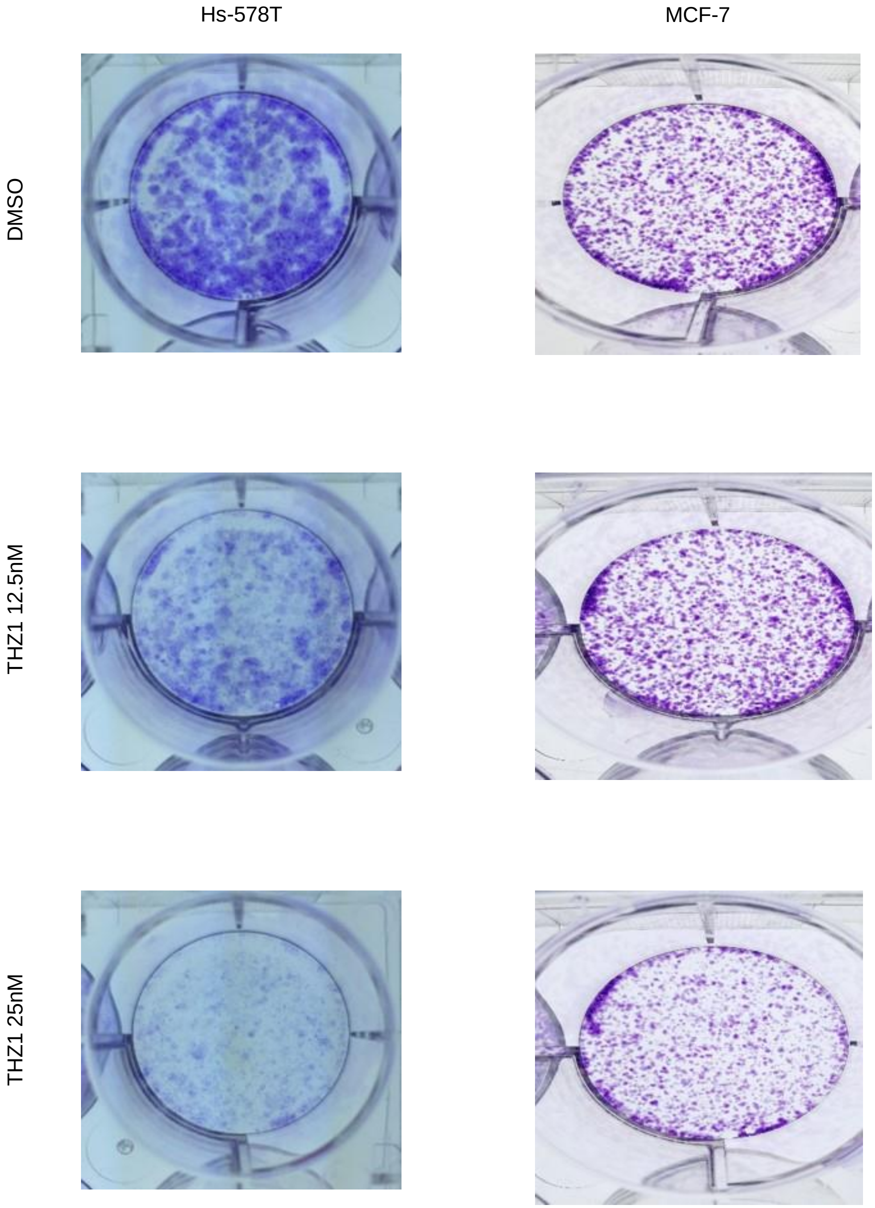

figure1D

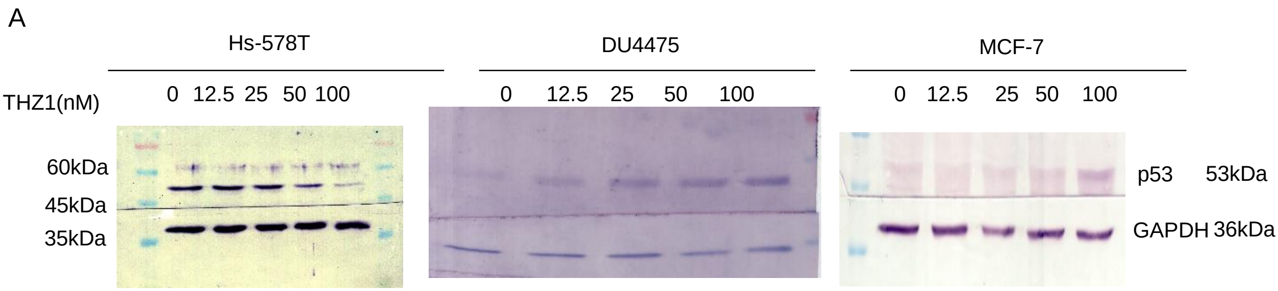
A
Hs-578T
DU4475
MCF-7
THZ1(nM)
0 12.5 25 50 100
0 12.5 25 50 100
0 12.5 25 50 100
60kDa
45kDa
35kDa
p53 53kDa
GAPDH 36kDa

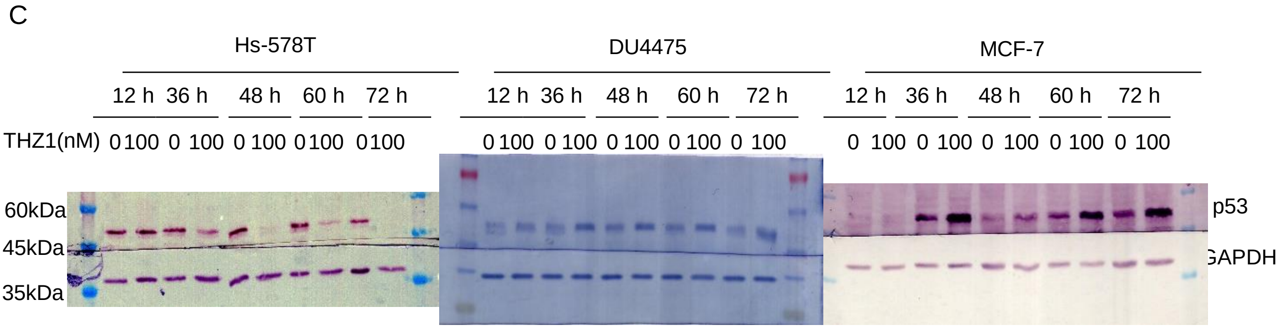
C
Hs-578T
DU4475
MCF-7
12 h 36 h 48 h 60 h 72 h
12 h 36 h 48 h 60 h 72 h
12 h 36 h 48 h 60 h 72 h
THZ1(nM) 0 100 0 100 0 100 0 100 0 100
0 100 0 100 0 100 0 100 0 100
0 100 0 100 0 100 0 100 0 100
60kDa
45kDa
35kDa
p53

figure2

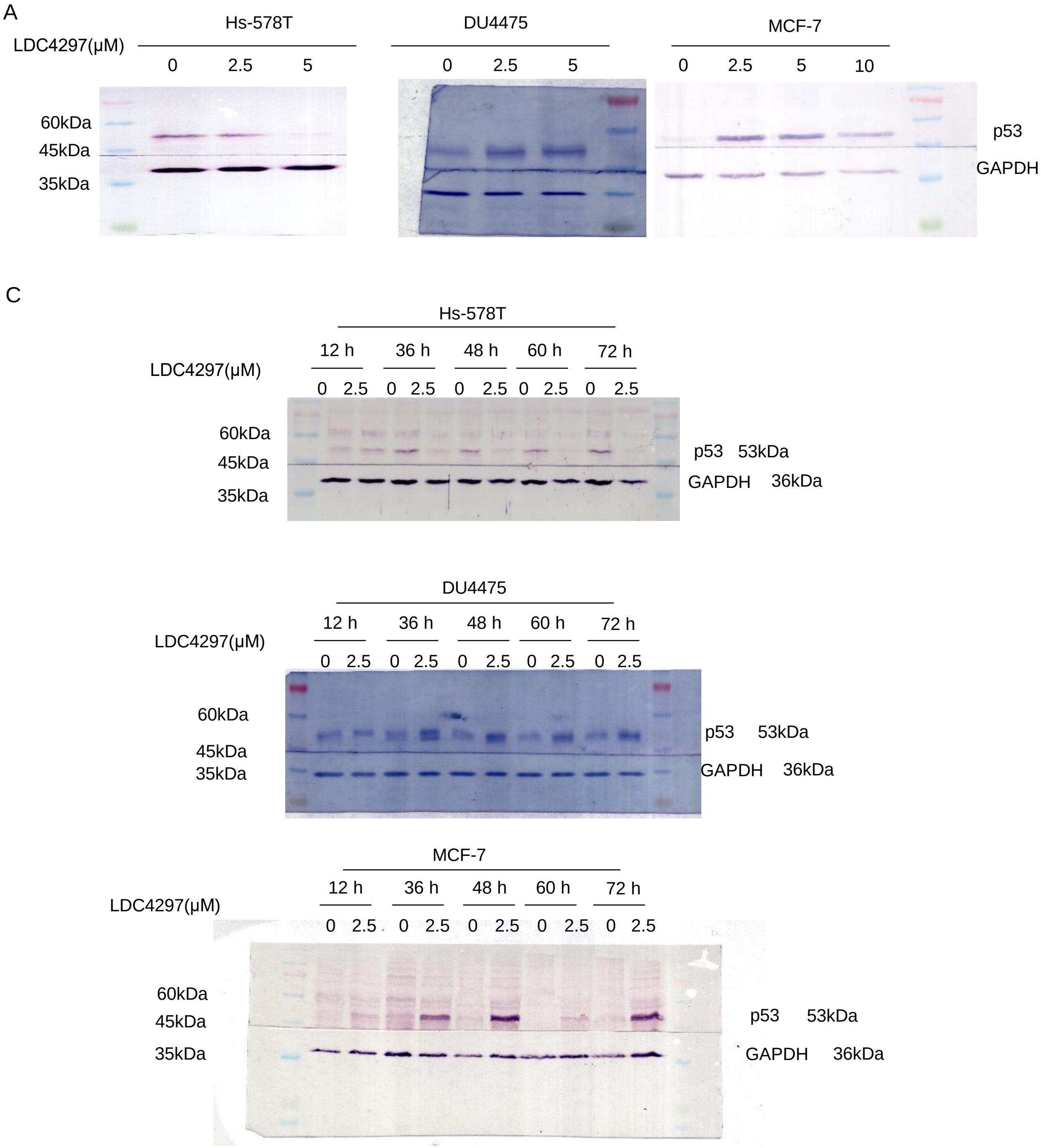
A
Hs-578T
DU4475
MCF-7
LDC4297(μM)
0 2.5 5
0 2.5 5
0 2.5 5 10
60kDa
45kDa
35kDa
p53
GAPDH
C
Hs-578T
LDC4297(μM)
12 h 36 h 48 h 60 h 72 h
0 2.5 0 2.5 0 2.5 0 2.5 0 2.5
60kDa
45kDa
35kDa
p53 53kDa
GAPDH 36kDa
DU4475
LDC4297(μM)
12 h 36 h 48 h 60 h 72 h
0 2.5 0 2.5 0 2.5 0 2.5 0 2.5
60kDa
45kDa
35kDa
p53 53kDa
GAPDH 36kDa
MCF-7
LDC4297(μM)
12 h 36 h 48 h 60 h 72 h
0 2.5 0 2.5 0 2.5 0 2.5 0 2.5
60kDa
45kDa
35kDa
p53 53kDa
GAPDH 36kDa

figure3

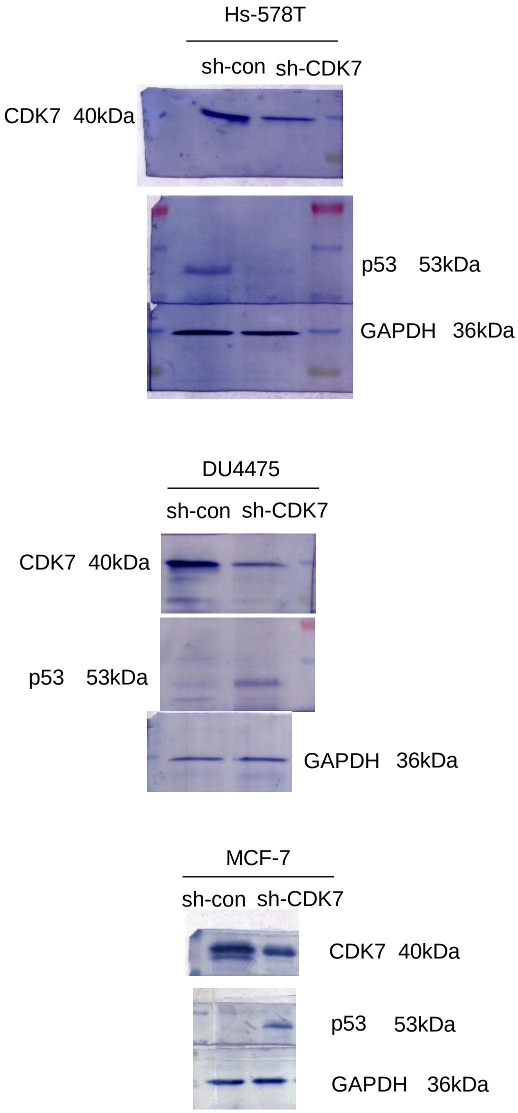
Hs-578T
sh-con sh-CDK7
CDK7 40kDa
p53 53kDa
GAPDH 36kDa
DU4475
sh-con sh-CDK7
CDK7 40kDa
p53 53kDa
GAPDH 36kDa
MCF-7
sh-con sh-CDK7
CDK7 40kDa
p53 53kDa
GAPDH 36kDa

figure4

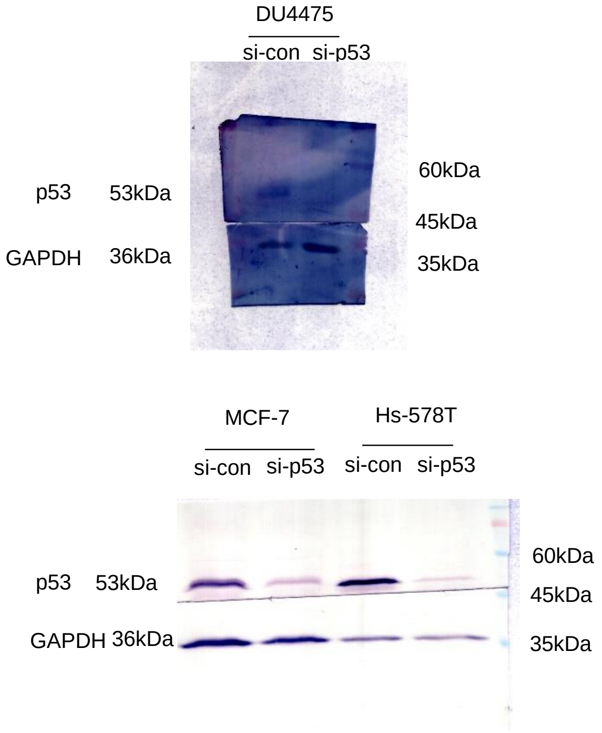
DU4475
si-con
si-p53
p53
53kDa
GAPDH
36kDa
60kDa
45kDa
35kDa
MCF-7
Hs-578T
si-con
si-p53
si-con
si-p53
p53
53kDa
GAPDH
36kDa
60kDa
45kDa
35kDa

figure5

The protein markers were purchased from
https://www.genefirst.com (Cat:GF6619)

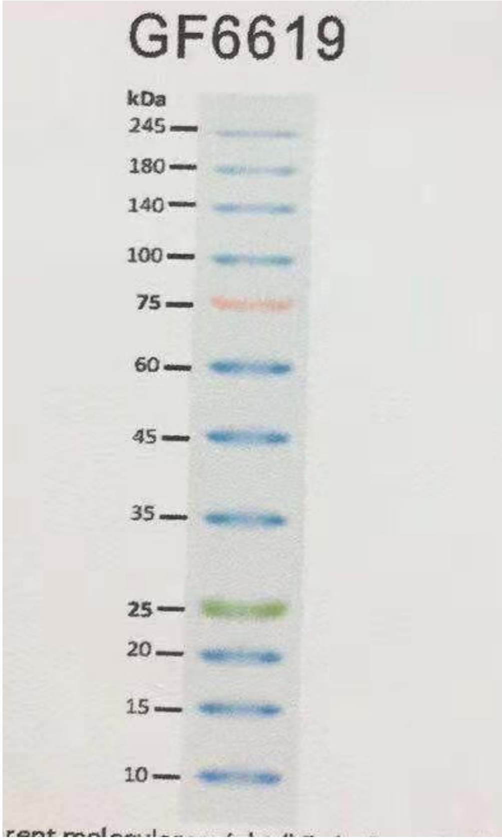

GF6619

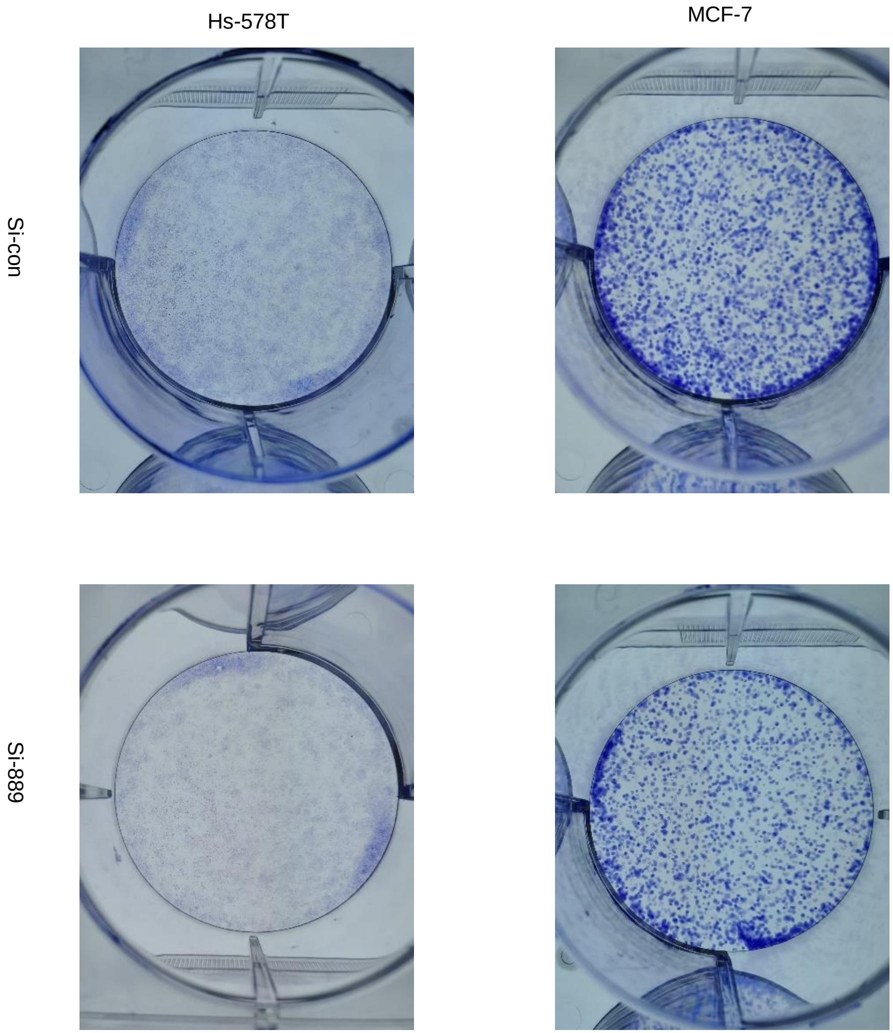

figure5E
